# Large-Scale Field Application of RNAi Technology Reducing Israeli Acute Paralysis Virus Disease in Honey Bees (*Apis mellifera*, Hymenoptera: Apidae)

**DOI:** 10.1371/journal.ppat.1001160

**Published:** 2010-12-23

**Authors:** Wayne Hunter, James Ellis, Dennis vanEngelsdorp, Jerry Hayes, Dave Westervelt, Eitan Glick, Michael Williams, Ilan Sela, Eyal Maori, Jeffery Pettis, Diana Cox-Foster, Nitzan Paldi

**Affiliations:** 1 United States Department of Agriculture (USDA), Agricultural Research Service (ARS), U.S. Horticultural Research Lab, Fort Pierce, Florida, United States of America; 2 University of Florida, Department of Entomology and Nematology, Gainesville, Florida, United States of America; 3 Department of Entomology, The Pennsylvania State University, University Park, Pennsylvania, United States of America; 4 Florida Department of Agriculture, Bureau of Plant and Apiary Inspection, Apiary Inspection Section, Division of Plant Industry, Gainesville, Florida, United States of America; 5 Beeologics Inc., Miami, Florida, United States of America; 6 Robert H. Smith Institute for Plant Sciences and Genetics in Agriculture, Virus Laboratory, Faculty of Agricultural, Food and Environmental Quality Sciences, The Hebrew University of Jerusalem, Rehovot, Israel; 7 USDA, ARS, Bee Research Laboratory, Beltsville, Maryland, United States of America; Stanford University, United States of America

## Abstract

The importance of honey bees to the world economy far surpasses their contribution in terms of honey production; they are responsible for up to 30% of the world's food production through pollination of crops. Since fall 2006, honey bees in the U.S. have faced a serious population decline, due in part to a phenomenon called Colony Collapse Disorder (CCD), which is a disease syndrome that is likely caused by several factors. Data from an initial study in which investigators compared pathogens in honey bees affected by CCD suggested a putative role for Israeli Acute Paralysis Virus, IAPV. This is a single stranded RNA virus with no DNA stage placed taxonomically within the family Dicistroviridae. Although subsequent studies have failed to find IAPV in all CCD diagnosed colonies, IAPV has been shown to cause honey bee mortality. RNA interference technology (RNAi) has been used successfully to silence endogenous insect (including honey bee) genes both by injection and feeding. Moreover, RNAi was shown to prevent bees from succumbing to infection from IAPV under laboratory conditions. In the current study IAPV specific homologous dsRNA was used in the field, under natural beekeeping conditions in order to prevent mortality and improve the overall health of bees infected with IAPV. This controlled study included a total of 160 honey bee hives in two discrete climates, seasons and geographical locations (Florida and Pennsylvania). To our knowledge, this is the first successful large-scale real world use of RNAi for disease control.

## Author Summary

High rates of honey bee mortality continue to threaten food security and apicultural industries worldwide. At least some of these losses are likely the result of viral infections. Application of RNAi technologies in the treatment and management of disease promises new solutions to disease problems through the naturally occurring biological processes of living organisms. This study applied a novel dsRNA product developed specifically with the aim of improving honey bee health. The results demonstrate the successful application of RNAi strategies to improve disease tolerance. Honey bees were fed a dsRNA product, Remebee-I, in the presence of the Israeli Acute Paralysis Virus. Treatment resulted in larger colony populations and thus increased honey production. We show that IAPV specific homologous dsRNA successfully curbed the negative effects of IAPV infection in 160 honey bee hives in two discrete climates, seasons and geographical locations (Florida and Pennsylvania). We provide the first successful demonstration of the use of RNAi as a preventative treatment for an insect disease on such a large scale.

## Introduction

The importance of honey bees as pollinators of crops to the global economy far surpasses their contributions in terms of honey production [1]. In all, 52 of the world's 115 leading agricultural crops rely on honey bee pollination to some extent. These crops represent approximately 35% of the human diet [2]. Insect pollination, which is provided predominately by honey bees, is estimated to have a value of US$ 212 billion [3]. Honey bee populations have been decreasing globally in recent years [4]. Since fall 2006, honey bees overwintering in the U.S.A. have faced unusually high rates of mortality, in part because of a phenomenon now known as Colony Collapse Disorder (CCD) [5]. Several hypotheses have been offered to explain CCD and existing and emerging pathogens have been implicated either directly or indirectly [6]. Colonies affected by CCD are infected with larger numbers of pathogenic organisms than control colonies, yet no single pathogen was found associated with all affected colonies [7]. In another effort, researchers did find that single-stranded RNA viruses, specifically picorna-like viruses, occurred at elevated levels in CCD colonies. These elevated levels of viruses may interfere with gene transcription, thus reducing immune response competence and pesticide detoxification capabilities, subsequently leading to premature death of infected bees [8].

Honey bees are susceptible to a host of picorna-like viruses, including the closely related Acute Bee Paralysis Virus (ABPV), Kashmir Bee Virus (KBV), and Israeli Acute Paralysis Virus (IAPV) [9], [10]. The latter of these three viruses was identified as a good marker for CCD in initial studies, especially when found in association with the microsporidia *Nosema* sp. [6]. While IAPV is probably not the sole cause of CCD [7], its ability to cause increased mortality in honey bees has been established [11].

The process of post-transcriptional gene silencing is thought to be an evolutionarily-conserved cellular defense mechanism used to prevent the expression of foreign genes and is commonly shared by diverse flora and phyla [12]. The presence of long double-stranded RNAs in cells stimulates the activity of a ribonuclease III, Dicer, which is involved in the processing of the double stranded RNA (dsRNA) into short interfering RNAs (siRNAs). The RNAi response also features an endonuclease complex, commonly referred to as an RNA-induced silencing complex (RISC), which mediates cleavage of target ssRNA having sequence complementary to the antisense strand of the siRNA duplex.[13], [14], [15], [16].

In a variety of organisms, exogenously applied dsRNA or their siRNA derivatives, can be used to arrest, retard or even prevent a variety of pathogens. In some of these organisms, such as plants and the nematode *C. elegans*, an amplification stage follows the initiation stage of gene silencing, involving an RNA dependent RNA Polymerase (RdRp), which may lead subsequently to degradation of RNAs outside the initial dsRNA region of homology [17]. RNAi can spread from the initial site of dsRNA delivery, producing interference phenotypes throughout the treated animal. To serve as a preventive or curative strategy, amplification and systemic spread of the silencing signal are both paramount. In some invertebrates, including honey bees, a systemic interference defective (SID) gene encodes a transmembrane protein that is an important participator in the systemic RNAi pathway. Apparently, these SID1-like proteins channel dsRNAs between cells, enabling systemic spread of the silencing signal [18], [19]. Although a canonical invertebrate RNA dependent RNA Polymerase (RdRP) homologue has not yet been described, there is evidence that such RdRp activity may occur via other enzymes, leading to amplification of the silencing signal in insects [20].

IAPV specific dsRNA (Remebee-IAPV or herein Remebee-I) was used successfully to prevent bees from succumbing to infection from IAPV in small scale lab experiments whereas bees fed Green Fluorescent Protein (GFP) dsRNA and virus died in a manner similar to the IAPV fed control bees [12]. Although these results were exciting per-se, transferring RNAi from a well characterized and efficient tool in the lab and making it successful in preventing the adverse effects of virus infection in the field, remains notoriously difficult.

We present the first large-scale real world successful use of RNAi for disease control. We attempted to determine if IAPV specific homologous dsRNA can be used to reduce impacts from IAPV infection in 160 honey bee hives in two discrete climates, seasons and geographical locations (Florida and Pennsylvania). To our knowledge, this is the first successful demonstration of the use of RNAi as a preventative treatment for an insect disease on such a large scale.

## Results

### Brief overall set up, feeding, and monitoring of bees, hive health, honey production

The field demonstration in FL was designed in a manner that permitted us to follow IAPV-infested bee colonies (some given Remebee-I and others not) for six weeks. One hundred standard colonies of honey bees were split into 5 groups with 20 colonies per group. Four groups were located within 100 m of one another (non-isolated) while a 5^th^ group was isolated from the remaining four by at least 3.2 km to measure any environmental effects due to location.

Treatment allocations (20 colonies per treatment) were as follows:

Treatment 1 – no treatment – non isolated

Treatment 2 – Remebee-I only – non isolated

Treatment 3 – Remebee-I+IAPV – non isolated

Treatment 4 – IAPV only (fed in sugar water solution) – non isolated

Treatment 5 – no treatment– isolated

### Treatment effects on colony strength parameters- Bee health

#### Brood area

This parameter, cm^2^ brood area, expressed as a cumulative percentage of coverage of each frame in a hive, was measured to assess the potential effects of Remebee-I on queen fecundity and can be used as a proxy to subsequent potential hive population (see Supporting Information S1). In Florida (FL), no significant difference was observed in the area of capped brood in Remebee-I treated versus non-treated controls (F_4,95_ = 1.2; P = 0.30). In Pennsylvania (PA), a BACI design analysis (see *Statistical Analysis*) was employed, and no significant difference in the change in brood area was identified between treatment groups (F_2,57_ = 0.05; P = 0.9495).

Remebee-I has no negative effect on honey bees. Remebee-I only without IAPV challenge was only applied in FL and not in PA. There was no significant difference in all parameters checked (total population, forager activity and total honey produced) between Remebee-I only treatment compared with non-treated, non IAPV challenged control hives. For continuity of figures thus this data is represented by the non IAPV challenged control hives.

#### Total population of adult bees

**Figure 1:**
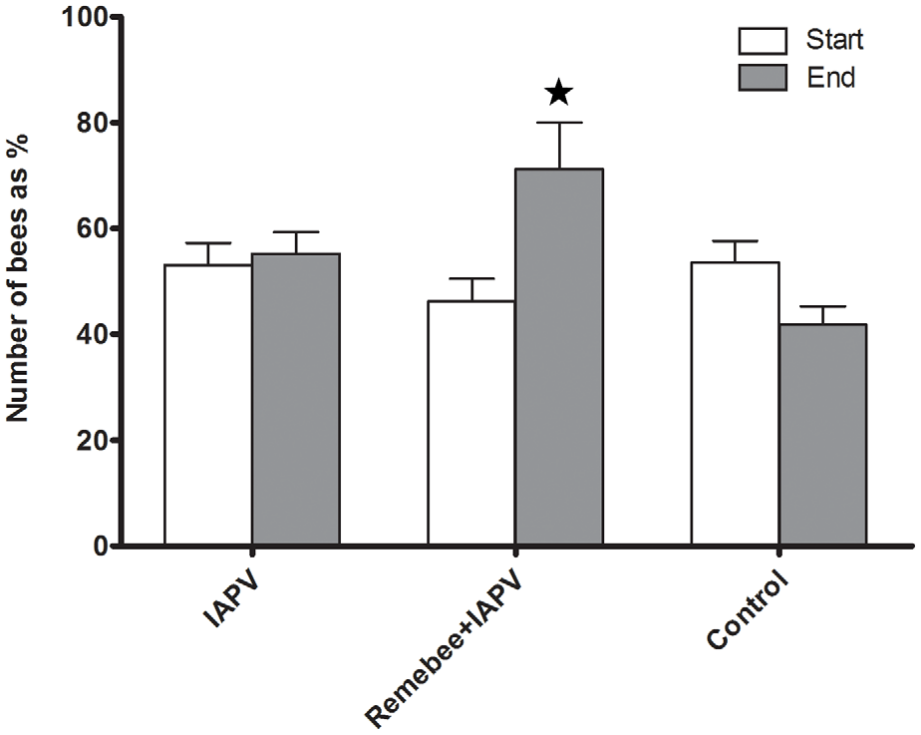
Change in the total bee population (Florida). The number of bees in the Remebee-I+IAPV was significantly different 5 weeks later (N = 40, p<0.002) compared to the control and IAPV alone treatments which were not significantly different (N = 40, p>0.25). There was no significant difference between treatments at start or at end points of the PA trial.

In the FL trial, a significant difference was detected between the final adult bee populations in colonies receiving Remebee-I+IAPV which were greater than colonies receiving any other treatment (Figure 1, F_4,95_ = 3.21; P = 0.0167). In the PA trial, the change of adult bee population over the course of the study did not differ significantly between treatment groups (F_2,57_ = 0.05; P = 0.9495).

#### Forager activity

Honey bees forage for nectar and pollen at different intensities, depending on the flowering resources. If the virus reduces the life-span of the adult bees, we would expect to see a reduced relative forager activity in the virus-inoculated groups. In FL, on three different dates after Remebee-I administration was terminated, returning foragers were counted at the colony entrance (approximately 2, 3 and 5 weeks after virus administration, Supporting Information S1). This was done simultaneously by several observers who moved between the colonies and counted returning foragers for one minute for each hive, and then rotated between the treatments to preclude any possibility of bias. Although immediately after virus infection there were no significant differences in forager counts, increasingly larger significant differences in forager activity were noted as the experiment progressed. In the first counting done 2 weeks after the virus administration, mean number of returning foragers per minute in the Remebee-I+IAPV was 37 (S.E.+/−2.4) and the comparable mean for the IAPV only group was 34 (S.E.+/−1.88) (t-test p>0.15 N.S.). However, 3 weeks post virus inoculation the mean number of returning foragers per minute in the Remebee-I+IAPV was 56 (S.E.+/−2.8), whereas it totaled only 43 for IAPV only (S.E.+/−2.8) (t-test, p<0.01). At five weeks post virus infection, the mean number of returning foragers per minute in the Remebee-I+IAPV was 37 (S.E.+/−1.8) yet only 22 in the IAPV only group (S.E.+/−1.6), (t-test, p<0.0001). This greater relative activity in the Remebee-I+IAPV treatment in relation to the IAPV only was somewhat correlated (rsqu = 0.35, p<0.0001) with the greater adult bee population in the Remebee-I+IAPV treatment in relation to the other treatments at the end of the experiment (Figure 1). In the PA site, extreme variability in returning forager counts, both at different times and between observers precluded the use of this data.

### Treatment effect on honey production

**Figure 2:**
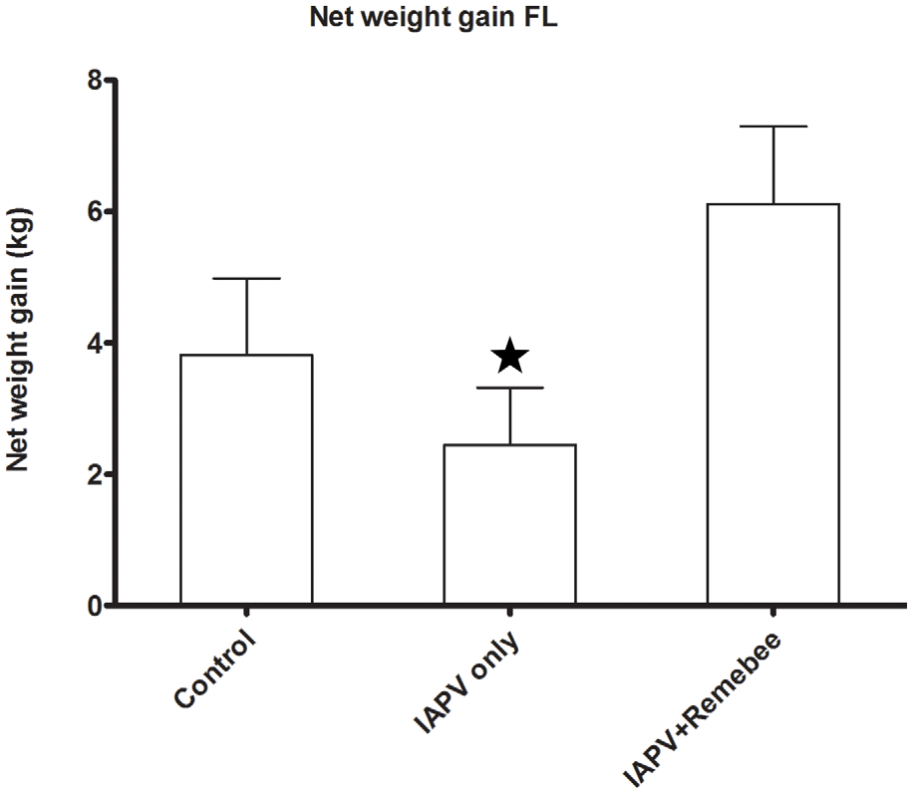
Net weight gain in hives (Florida). Total weight gained by the hives in the different treatments corresponds mainly to amount of honey collected. Remebee-I+IAPV hives produced significantly more honey compared with IAPV only treated hives (N = 40, p<0.03). Honey production in control colonies and IAPV colonies were not significantly different (N = 40, p>0.2).

**Figure 3:**
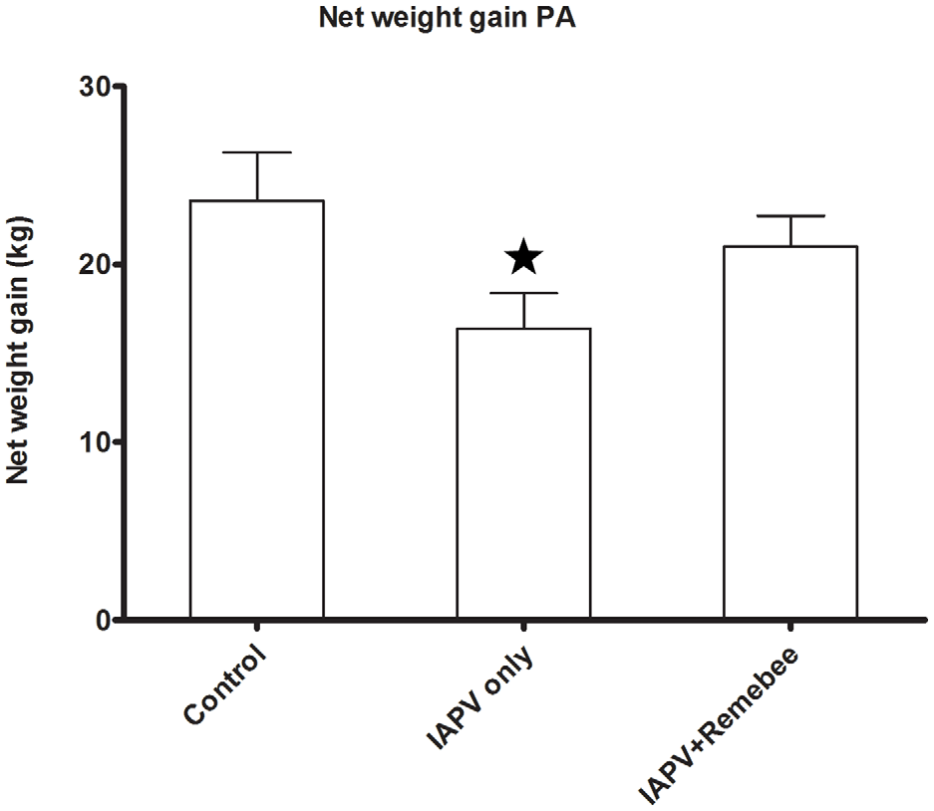
Net weight gain in hives (Pennsylvania). Total weight gained by the hives in the different treatments corresponds mainly to amount of honey collected. The difference between the weight at the start and the end of the experiment (4 months) shows that the non infected controls (mean = 23.5kg) and the Remebee-I+IAPV (mean = 21kg) made significantly more honey than hives receiving IAPV alone (mean = 16.3kg) (F = 2.7; df = 4.92; P = 0.034).

Honey (or net weight gain) is the ultimate proxy to the total active population of the hive. The non treated control produced the most honey in PA, but not in FL. In FL, colonies treated with Remebee-I+IAPV produced significantly more honey than colonies receiving IAPV alone (Figure 2, N = 40, p<0.03). In PA, the difference between the weight at the start and the end of the experiment (4 months) shows that the non infected controls gained the most weight (mean gain = 23.5kg), whereas Remebee-I+IAPV had gained slightly less (mean = 21kg). Both made significantly greater weight gains compared with the group receiving IAPV alone (mean = 16.3kg) (Figure 3 F = 2.7; df = 4.92; P = 0.034).

### IAPV specific siRNAs are produced by Remebee-I treatment

Subsequent trials done under a similar protocol were repeated in the winter of 2009–10 in FL and in California (CA). Samples of bees were collected just before IAPV challenge and 2-weeks post treatment. Northern analysis was done with IAPV specific sequence probes corresponding with the Remebee-I sequence. The results of these are presented in detail in Supporting Information S2. High levels of discrete Dicer Remebee metabolites are evident in Remebee-I treated hives prior to IAPV challenge up to four weeks after a Remebee-I application. Non- Remebee-I treated bees are mostly negative, but a low signal was detected in some colonies. Subsequent to IAPV challenge, levels of siRNAs and IAPV metabolites are highly elevated in both Remebee-I and in non-Remebee-I treated hives, showing that production of dsRNA is a natural defense mechanism in bees against IAPV infection.

*Varroa* levels are unaffected by treatment. The change in varroa prevalence on adult bees did not differ significantly between treatment groups in either the FL (F_4,95_ = 2.39; P = 0.056) or PA (F_2,57_ = 1.03; P = 0.3642) trials.

**Figure 4:**
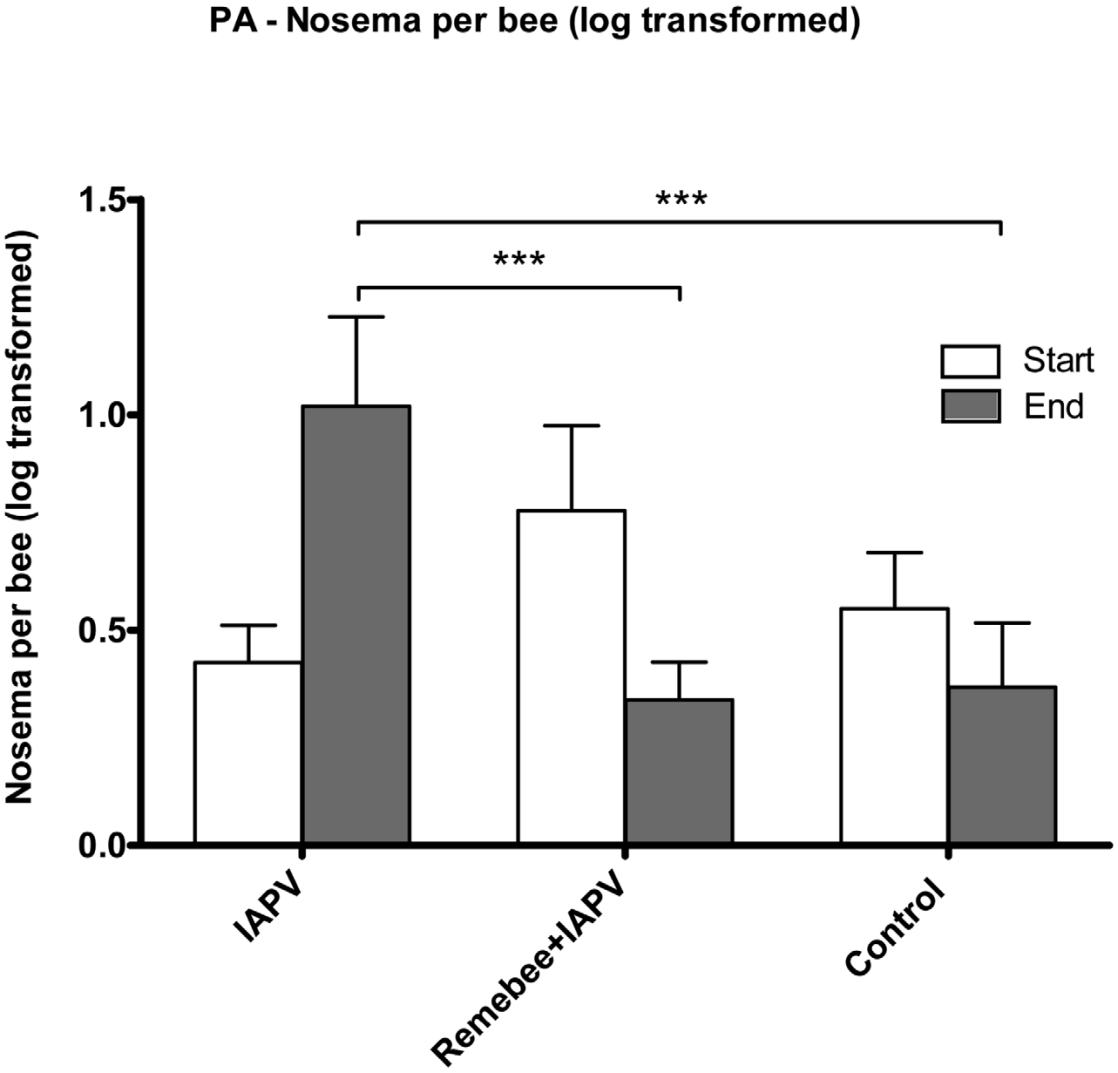
*Nosema* spore counts per bee. *Nosema* spore counts at the 4 weeks and at the end point of the Pennsylvania trial. At 4 weeks after virus introduction, IAPV only and Remebee-I+IAPV Nosema spore counts were elevated in contrast to the uninfected control, but insignificantly so because of the large variance. However, 8 weeks later, levels of *Nosema* spores were significantly greater in the IAPV only group (1.02 million per bee) relative to the uninfected control (0.36 million per bee) (N = 31, p<0.006). Remarkably, spore levels in the treatment group receiving Remebee-I+IAPV (0.33 million per bee) were not significantly different from the uninfected control (N = 30, p>0.89), but were significantly different from the IAPV only cluster (N = 27, p<0.007). BACI design; Proc mixed = repeated measured analysis with unstructured covariance matrix. No effect of treatment over time. Df = 4, 95; F = 0.47; P = 0.7586.

*Nosema* levels: While the change in nosema levels in the FL trial was not significantly different between treatment groups (F_4,95_ = 0.47; P = 0.7586), a highly significant difference in nosema spore levels was detected at the end of the PA trial (F_2,57_ = 8.62; P = 0.0005) (Figure 4). Indeed, within the duration of the experiment, nosema spores levels increased in the IAPV treated group, yet went down in both Remebee-I+ IAPV and uninfected control colonies.

A brief summary of findings is presented in Table 1.

**Table 1:** Summary of Remebee-I treatment on honey bee health and mortality.

Effect of RNAi treatment	Summary of the effects of treatment with Remebee-I challenged with IAPV in contrast with control treatment (IAPV challenge only)	
	Florida	Pennsylvania
Brood	No effect	No effect
Levels of specific siRNAs	Highly elevated	Not checked
Mean bee population per hive at the end of the trial	Greater in the Remebee-I treated group (p<0.002)	Not significantly different
Mean adult forager activity	Progressively greater difference (Starting with 8% N.S., through 30% p<0.01, and finally 68%, p<0.0001)	Measurement variability too broad undetermined
Total weight gain (honey)	Greater in the Remebee-I treated group (p<0.03)	Greater in the Remebee-I treated group (p<0.034)
Nosema levels	Not significantly different	2.5 times lower in the Remebee-I group (p<0.007)
Varroa counts	Not significantly different	Not significantly different

## Discussion

### Effects of IAPV on bees

The negative effects of IAPV on honey bee health and colony vigor is evidenced by lower honey weight gains (Figures 2 and 3). Environmental factors and terrain also influence the availability of forage and thus can seriously reduce or increase the effects of virus infection on a hive by altering foraging patterns. Large scale examination of the RNAi treatment under such varied conditions at two separate and diverse locations (i.e. FL and PA) was challenging. However, one would expect that 7–8 weeks after a young queen begins to oviposition prolifically (see Supporting Information S1 for brief overview), all hives would be overflowing with bees. This was evidently not the case in this situation, and the IAPV only group reversed in total population and bee/brood ratio. Thus, we observed relative de-population of the hive with probable greater loss of foragers in IAPV only infected hives. We conducted the trials described herein in spring and summer, whereas CCD is a mostly winter phenomenon [1], [7]. This may be the reason that we did not see many hives devastated by CCD. However, subsequent trials performed in the 2009–10 winter in Florida and California resulted in 40% and 60% collapse of hives, respectively (Hunter Wayne. and Oliver Randy., personnel communication). Beyond cold weather providing additional stress on the bees, some of the difference may be attributed to the viruses' suppressors of gene silencing. In plants, these viral suppressors of gene silencing have more optimal enzymatic kinetic coefficients under cold temperatures in relation to the silencing enzymes, often leading to more acute virulence [21]. This could help explain the initiation and overall devastation of hives in the U.S. following winter cold snaps (Dennis VanEngelsdorp unpublished observations).

### Effects of Remebee-I protecting bees from IAPV

Our hypothesis that Remebee-I would protect bees from IAPV infection was supported by multiple observations: First, in FL, the Remebee-I+ IAPV treated hives were the only colonies with significantly increasing numbers of bees during the study. In PA bees increased within all treatments with no significant differences. Some of the difference between the two trials could account for this difference in observations. In PA the virus was introduced twice into the colonies within a three day period (instead of once), and the total amount of virus introduced was thus much higher than the FL trial. The PA trial starter hives were weaker in strength, and had more collapses of hives across all treatments prior to infection than those starting the FL trial. Second, although bee to brood ratios started lower in Remebee-I treated hives it became stronger and remained so until the end of the trial. The change in the ratio is attributed to changes in the adult bee counts, since the capped brood coverage was the same between treated and non-treated bees, thus these hives also contained more adult foragers, which resulted in significantly more honey production over the IAPV only treatment (Figure 2,3).

Furthermore, in subsequent trials, molecular evidence now proves that Remebee-I is active in the Remebee-I + IAPV treated groups, as determined by the presence of siRNA (see Supporting Information S2). The strong presence of siRNAs probably restricts the severity of the disease in the bees leading to a longer life-span and subsequently to an overall greater number of bees, with more foragers and consequently a greater yield of honey. It is interesting to note the natural occurrence of these siRNAs in bees receiving IAPV challenge. Presence of these siRNA in non- Remebee-I treated hives prior to infection may be a result of natural virus infection prior to the challenge, or by transcription of integrated viral sequences in the bee genome [22].

#### Honey production

The most obvious measure of bee hive health is honey production. While overall honey production was not on the levels of commercial production, due to the remote locations and time of year, the Remebee-I + IAPV treated hives still produced 30–300% more honey than the IAPV-only treated hives in PA and FL respectively (Figures 2,3).

#### Natural modes of infection

Some bees in hives which were neither treated with Remebee-I nor fed IAPV eventually became infected with virus. There are several ways this may have occurred. First, healthy bees and IAPV-infected bees may forage on the same flowers, thus facilitating pathogen transmission [7], [23], [24]. Secondly, bees may drift randomly between colonies. Finally, viruses may be transferred through the residual varroa mites [25]. Foragers from the Remebee-I + IAPV treatment may have been protected from acute viral disease because they were chronically infected with virus at very low levels, thus failing to develop full symptoms and remaining apparently healthy. This may be due to the virus being introduced together with Remebee-I, which serves as a template for dsRNA/siRNA amplification, thereby providing continued silencing of the virus. The recent results of the dramatic amplification effect of the virus on the siRNAs demonstrate this clearly (Supporting Information S2).

#### Remebee-I safety to bees

Remebee-I was shown to be active in the Remebee-I+IAPV treated colonies by the presence of siRNA in treated bees (Supporting Information S2). The high titers of siRNAs probably restrict the severity of IAPV in bees. The protection provided by Remebee-I appears to last throughout the lifespan of an individual bee. Thus, after several weeks without the application of Remebee-I to colonies, newly emerged bees which were not fed the treatment may not be protected. Notwithstanding, some evidence points to the possibility of providing protection from virus to the adult bees through larval feeding by the nurse bees [26].

#### *Varroa* mites

*Varroa* mites are arguably one of the largest challenges to beekeepers. However, prevention of *Varroa* is intensively practiced by beekeepers using a variety of miticides and by application of best management practices. While *Varroa* levels were found at equal levels in CCD and non-CCD colonies at the time of sample collection, coumaphos, a miticide commonly used by beekeepers, was found at higher levels in non-CCD versus CCD colonies. This may suggest that non-CCD colonies had mite levels more aggressively controlled some time prior to sample collection, and the increased viral loads in CCD colonies is a legacy effect of different mite levels some time before sample collection [27]. Indeed, most ubiquitous bee viruses, with IAPV amongst them, have been found in the *Varroa* mite and transmission has been demonstrated [24], [28].

#### CCD multi-pathogen syndrome

The evidence which ties virus with nosema to CCD continues to increase. Nosema is one of the most prevalent adult honey bee diseases and is caused by two described species of microsporidia (*Nosema apis* and *Nosema ceranae*, *N. apis* and *N. ceranae*, respectively). Researchers from Spain showed that natural *N. ceranae* infection can induce the sudden collapse of bee colonies, establishing a direct correlation between *N. ceranae* infection and the death of honey bee colonies [29]. *Nosema ceranae* has existed in honey bees within the United States since at least 1996 [30] without any reported dramatic colony declines until recently. However, an association between viruses (IAPV and others) with *Nosema* in CCD colonies has been established [7], [11]. Our data further supports this association. In the PA trial, colonies treatment with Remebee-I, which reduced virus levels, may have led to a concurrent reduction in nosema levels comparable to that of the control non-virus inoculated colonies (Figure 4), further supporting the association of acute virus disease and elevated *Nosema* levels in hive collapse. Recently it was shown that an RNAi strategy against *Nosema* is also efficacious [31], so using RNAi to target both viruses and *Nosema* in concert is now feasible.

We postulate that foragers from Remebee-I + IAPV treated hives were protected from this acute viral disease, because the siRNAs they produced protected them and enabled chronic infection with virus at very low levels. These bees thus failed to develop symptoms and remained sufficiently healthy to support longer forager activity. This may be due to the concurrent presence of virus together with Remebee-I whereby the replicating viral genome serves as a template for dsRNA/siRNA amplification *via* virus or host encoded RdRp that may be amplifying the silencing signal.

### Conclusions

IAPV specific dsRNA (Remebee-I) was used successfully to prevent bees from succumbing to infection from IAPV. The results further demonstrate the possibility to produce targeted treatments for bee pathogenic diseases. These field results demonstrate the successful application of dsRNA as a viable treatment to solve a real world problem, which may further lead to concerted efforts to utilize this ubiquitous natural mechanism, RNAi, for the benefit of the bees, beekeepers, and hopefully to other applications in agriculture and veterinary health.

## Materials and Methods

To determine if IAPV can be silenced using RNAi technology, we had to (1) purify IAPV from honey bees, (2) infect honey bee colonies with IAPV and/or Remebee-I and (3) determine IAPV presence in experimental colonies.

### dsRNA synthesis

Essentially as described in [11].

### IAPV purification

Approximately 40 adult forager honey bees were collected from 10 colonies in a Florida bee yard (apiary) where CCD had been reported. Each bee was processed individually and tested using rtPCR for the presence of Israeli Acute Paralysis Virus (IAPV), genome – NC_009025; Acute Bee Paralysis Virus (ABPV) genome – NC_002548; Kashmir Bee Virus (KBV) genome – NC_004807; Black Queen Cell Virus (BQCV), genome-NC_003784; Deformed Wing Virus (DWV) genome NC_004830. All bees had more than one virus detected so inoculum was prepared from bees which tested positive only for IAPV+KBV by homogenizing the bees with glass beads in small amounts of 10 mM buffer phosphate, pH 7.2 containing 0.02% DETCA (Sigma-Aldrich Cat #22,868-0). Inoculum was prepared by passing the virus solution through a syringe filter, 0.45 µm, to remove bacteria, after which ∼10 µl were administered by microinjection along the lateral side of the abdomen of ∼700 pupae using a Hamilton syringe with a 30Gx½ gauge sterile needle. Inoculated pupae were kept in petri dishes covered with slightly damp filter paper and maintained at 21–23°C for three days to permit virus replication.

### Virus purification

On the third day, batches of about 50 pupae were homogenized with glass beads. Small amounts of 10 mM buffer phosphate (pH 7.2 contained 0.02% DETCA, Sigma-Aldrich Cat #22,868-0) were added to the homogenates. The homogenates were collected in a beaker volume adjusted to ∼350 ml with buffer (see above) and mixed. Each sample was split into two 250 ml centrifuge tubes and centrifuged at 300×g (∼1,400 rpm) on a GSA rotor for 20 min. The supernatant (S1) was collected and kept at 4°C for 3 d. The pellet (P1) was recovered and saved at 4°C. Since some precipitation was noticed after 3 d, the supernatant was centrifuged again as before for 10 min to remove debris. Supernatant (S1) next was transferred to 12 ultracentrifuge tubes (about 26 ml/tube) (Beckman Cat #355618) and centrifuged for 4 h, 4°C, at 37,000 rpm (∼124,500×g) (Beckman Type 50.2 Ti rotor, Beckman Optima L-70K Ultracentrifuge). After 4 h the supernatant (S2) was removed and saved. The pellet (P2) was resuspended in 10 mM phosphate buffer containing 4% Brij 58 (Aldrich Cat #388831) and 0.4% Sodium deoxycholate (Sigma-Aldrich D6750): about 1 ml of buffer was used per tube. It was necessary to insert a spatula to help pellet into solution and this was followed by vortexing the suspension. The content from each tube was transferred to clean 50 ml centrifuge tubes. The process was repeated twice but only buffer phosphate was added the final time. Because the final solution was very thick, buffer was added to increase the final volume to ∼30 ml and this was mixed by inversion. The tube was centrifuged for 15 min at ∼10°C, 800×g (Beckman Coulter Allegra 25R), to remove debris. The pellet (P3) was saved at 4°C. (the pellet saved as a backup). The supernatant (S2) was transferred into two clean 50 ml tubes and 13.2 g CsCl (Amresco Cat #0415) were added to each tube. To ensure the right CsCl concentration, 13.2 g CsCl were added to ∼10 g sample; however the final volume was adjusted to 24 ml with buffer and gently mixed (up/down). The second tube was set by adding CsCl to the remaining sample. This final preparation was transferred to two ultracentrifuge tubes ∼25 ml (Beckman Cat #355618) and centrifuged at 37,000 rpm (∼124,500×g), 18°C for 24 h. After 24 h centrifugation, the tubes were removed carefully from the rotor and the whitish virus band collected by insertion of a needle attached to a syringe. Two more fractions were recovered for analyses: (1) the “liquid” part left after removing the virus band and (2) the “pellet” (P4) attached to the bottom of tube: Each fraction was transferred to dialysis tubes (Thomas Scientific Cat #3787-F42) and dialyzed overnight against nanopure filtered water followed by 3–4 additional changes in water the following day. After dialysis, content from the tubes was collected in 15 ml clean tubes and the volumes were measured. A subsample of 20 µl from each fraction was tested for virus presence.

### RNA extraction

Adult bees were transferred to 1.5 ml centrifuge tubes. Tri Reagent (Sigma Cat #T9424), was added and individual bees were homogenized in 0.5 ml Tri reagent using disposable pestles and glass beads. Homogenates were frozen at −20°C if needed. Samples then were centrifuged 10 min at 12,000×g, at 4°C. The clear supernatant was transferred to a new tube and left at least 5 min at room temperature (RT). Next, 0.2 ml chloroform was added and samples were shaken vigorously. This was followed by a10–15 min incubation at RT. Tubes were centrifuged 15 min at 12,000×g at 4°C. The colorless upper aqueous phase was transferred to a new tube and 0.5 ml isopropanol was added. After mixing, samples were allowed to stand for 10 min then spun 10 min at 12,000×g at 4°C. The supernatant was removed and the pellet containing the RNA was washed with 1 ml 75% ethanol. After 5 min centrifugation at 7,500×g at 4°C, the RNA was allowed to dry (5–10 min) and reconstitute in ∼30 µl Nuclease free water (Qiagen). RNA concentrations were measured in a Nanodrop, ND-1000 Spectrophotometer. Samples were diluted in Nuclease free water.

### Overall set up, feeding, monitoring of bees, hive health, and honey production

The field demonstration in FL was designed in a manner that permitted us to follow IAPV-infested bee colonies (some given Remebee-I and others not) for six weeks. One hundred standard colonies of honey bees were split into 5 groups with 20 colonies per group. Four groups were located within 100 m of one another (non-isolated) while a 5^th^ group was isolated from the remaining four by at least 3.2 km to measure any environmental effects due to location.

Treatment allocations (20 colonies per treatment) were as follows:

Treatment 1 – no treatment – non isolated

Treatment 2 – Remebee-I only – non isolated

Treatment 3 – Remebee-I+IAPV – non isolated

Treatment 4 – IAPV only (fed in sugar water solution) – non isolated

Treatment 5 – no treatment– isolated

Colonies were equalized according to standard protocols prior to the beginning of the study (frames of bees/brood moved between colonies until populations leveled) and were managed optimally for honey production. Data collected at the beginning, middle, and end of the study included: frames of adult bees, cm^2^ brood, the presence of other bee maladies (nosema, varroa, and tracheal mites), bee activity, honey production and IAPV presence/absence and titer. The study lasted 6 weeks from the date of colony inoculation with IAPV and was replicated in PA with the following modifications: Only Treatment groups 1, 3 and 4 were established and the trial lasted 12 weeks after inoculation to enable the bees to take advantage of a honeyflow (Tables 2 and 3). In PA the virus was introduced twice into the colonies within a three day period (instead of once as in FL), and the total amount of virus introduced was thus much higher than the FL trial. We calculated all test/sampling dates below from the date of the last treatment with Remebee-I. Controls 1, 2, and 5, accounted for ‘within treatments’, a Remebee-I alone treatment to evaluate any potential detrimental effects to bees, and a distant control to measure environmental effects in the absence of IAPV.

**Table 2:** Treatment Groups.

Florida- April to June 2008	Pennsylvania-May to October 2008
No treatment	No treatment
IAPV one application	IAPV two application
Remebee + IAPV (one IAPV application)	Remebee + IAPV (two IAPV application)
Remebee only	
No treatment - Isolated Control group	

**Table 3:** Treatment schedule for Florida and Pennsylvania locations.

Adams Ranch - Florida		Sligo- Pennsylvania	
Date	Activity	Date	Activity
April 22	Feeding	May 30	Feeding
April 24	Feeding	June 03	Feeding
April 29	Feeding+IAPV introduction	June 06	Feeding
May 02	Feeding	June 10	Feeding+IAPV introduction
May 06	Analysis (mp)+Feeding	June 13	Feeding+IAPV introduction
May 23	Analysis	June 17	Feeding
June 10	Analysis (ep)	June 20	Analysis (mp)
		July 21	Analysis
		Oct 03	Analysis (ep)

^(mp)^ – mid-point analysis includes weighing the hives and full bee assessment.

^(ep)^ – end-point analysis includes all activities of mid-point and preparation for hives disposal.

#### Frames of adult bees

This parameter was measured only at weeks 2.5 and 5. Two observers estimated (in tenths) the amount of frame area covered by bees for every frame in each colony. The scores from both observers were averaged and converted to number of bees per colony [32]. Estimates as % of total frame. Each frame is raised and the observer notes percentage coverage on both sides of the frame. If all the frame side is completely covered with bees, the observer scores 1, and if there are no bees on the frame side, he scores 0. Any other coverage is estimated by the observer from 0 to 1 in whole numbers. Two trial observers went through the hives independently, both at interim and final dates. [33]

#### Total brood area

This parameter was measured only at weeks 2.5 and 5. Two observers estimated total capped brood as % frame coverage. Each frame is raised and the observer notes % coverage on both sides of the frame. If all the frame side is covered with capped brood, the observer scores 1, and if there is no capped brood at all, he scores 0. Any other coverage is estimated (in tenths) the amount of brood (of any stage in development) covering both sides of a single frame for every frame in each colony. Since a queen excluder was placed between the deep and the honey super, there was no brood in the honey super at the final date. The scores from both observers were averaged and converted to cm^2^ brood based on the observation that one deep Langstroth comb (both sides) = 1754 cm^2^ [33].

#### Honey production

Deep and Super boxes were weighed in kilograms. Prior to introducing the super on top of each deep, the super with the empty frames was weighed in the field and marked.

Deeps were numbered 1–100 according to hive numbers, whereas supers were numbered 101–200, respectively. At the final end point both deeps and supers were weighed again, and the net gain was recorded. In PA, to determine the amount of honey produced by each colony, empty supers were weighed empty and added to colonies prior to any nectar flow. They were added to colonies as needed throughout the study. Due to delayed flower bloom the weights were taken as scheduled, but then again 4 weeks later to adjust for environmental differences between PA and FL seasons.

#### Bee activity counts

Hive activity at all colonies was determined at weeks 0, 2.5 and 5 by having multiple observers simultaneously counting the number of bees landing at the hive entrance for 60 seconds, for all hives in all treatments (Supporting Information S1).

#### Presence of other bee maladies

Samples of 300 bees were placed in 70% ethanol on 2 April (before any feeding with Remebee-I and/or virus) and at the end of week 5. The samples were used to determine initial and ending nosema, varroa mite, and tracheal mite loads. Samples were analyzed at the University of Florida Honey Bee Research and Extension Lab.

#### Feeding regimes

After feeding colonies once with 0.5 L of sugar water (66% sugar by volume) in glass feeding jars to ascertain that all hives were consuming the food, another five (FL) or six (PA) consecutive feedings of 0.5 L were given to colonies over a 2–3 week period. Thereafter, an empty honey super was added to all colonies and no further feedings occurred.

1. No treatment = Five or six feedings of 0.5 L sugar water per hive (FL and PA).
2. IAPV only treatment = In FL: five feedings of 0.5 L sugar water per hive, with feedings three, four, and five containing 500 micrograms IAPV per hive, and two of only sugar water. In PA: five feedings of 0.5 L sugar water per hive, with both feedings four and five supplemented with 500 micrograms of virus per hive.
3. Remebee-I + IAPV = Five or six feedings with 0.5 L sugar water supplemented with 10 mg Remebee-I per hive per feeding, with feeding three (FL) or both feedings four and five (PA) having an addition of 500 microgram purified IAPV per hive, per application.
4. Remebee-I only treatment = Five or six feedings with 0.5 L sugar water supplemented with 10 mg Remebee-I per hive per feeding (FL only).
5. Remote Control = The isolated untreated control of 20 hives was placed ∼1.8 miles due East from the test site, and provided five feedings of 0.5 L of sugar water per hive (FL only).

### Statistical analysis

Colonies were equalized at the beginning of the studies, starting and ending colony strength parameters were compared using ANOVA recognizing treatment as the main effect (PROC GLM). Honey gains in treated colonies in FL and PA were compared identically. However, to compare colony size measures in the PA trial and for levels of nosema and varroa mites, a Before-After Control-Impact (BACI) design [33], [34] was used. A BACI design us a way of comparing data that are measured before treatment with data obtained after treatment. In general, it can be described as a repeated measures analysis of variance (ANOVA) which is performed using colonies as replicates and the covariance structure that best suits the data (PROC MIXED, SAS Institute). Each variable is measured at the start of the experiment to show existing conditions before treatment and then after a treatment. The analysis then looks at whether the change in variable measures was different between treatment groups. A repeated measures analysis of variance [34] was performed using colonies as replicates and an unstructured covariance structure was performed using SAS statistical software (PROC MIXED) [35].

### Accession numbers mentioned in text

Israeli Acute Paralysis Virus (IAPV) genome – NC_009025 (RefSec); Acute Bee Paralysis Virus (ABPV) genome – NC_002548 (RefSec); Kashmir Bee Virus (KBV) genome – NC_004807 (RefSec); Black Queen Cell Virus (BQCV), genome-NC_003784 (RefSec); Deformed Wing Virus (DWV) genome NC_004830 (RefSec); RNA dependent RNA Polymerase protein (*C. elegans*)– NP_492131 (RefSec); SID-1 protein (*A. mellifera*) – XP_395167 (RefSec); GFP nucleotide sequence – U87625 (GenBank).

## Supporting Information

Supporting Information S1 Bee hives, feeding unit and field set up.
(0.43 MB PDF)

Supporting Information S2 Detection of siRNAs in bee samples.
(0.99 MB PDF)
