# Supplementary material for: Large-Scale Field Application of RNAi Technology Reducing Israeli Acute Paralysis Virus Disease in Honey Bees (Apis mellifera, Hymenoptera: Apidae)
Source: PLoS Pathog. 2010 Dec 23;6(12):e1001160. doi: 10.1371/journal.ppat.1001160 (PMC3009593; doi:10.1371/journal.ppat.1001160)

Large-scale field application of RNAi technology reducing Israeli Acute Paralysis Virus disease in honey bees (Apis mellifera, Hymenoptera: Apidae)

Wayne Hunter[1*], James Ellis[2], Dennis vanEngelsdorp[3*], Jerry Hayes[4], Dave Westervelt[4], Eitan Glick[5], Michael Williams[3], Ilan Sela[6], Eyal Maori[6], Jeff Pettis[7], Diana Cox-Foster[3], Nitzan Paldi[5*]

Supporting Information S1:

Brood rearing in honey bees- a quick overview

Both in Florida and Pennsylvania, capped brood area was estimated by two observers independently, and the mean of the two observers was calculated. This was done by estimating the % cover of capped brood on both sides of the 9-10 frames in the brood box super where the queen lays eggs. This is either totaled as a score of 0 (no brood) to 10 (100% capped brood cover on all frames), as was done in Florida, or calculated as a percentage of the total coverage 0-100%, as was done in Pennsylvania (Obviously, there is no difference between these). The total coverage is then the simple composite sum of the estimates. Since the brood box ("Deep") has 10 frames, each with two sides scored, the theoretical maximum coverage is 200, and the minimum is zero.

The queen bee lays eggs inside cells at varying rates. This fluctuates between locations and seasons, and is a function of forage availability, hive well-being, queen genotype and seasonal weather. The hive bees then tend to the young larvae that emerge and feed them prolifically. After several days the bees fill the cell with food and cap the cell. This capped brood constitutes 70-80% of the total brood area, but is much easier to measure visually. Bees emerge from the capped cells after undergoing metamorphosis (larva→ pupae and then→ adult). Premature death of the adult foragers, leads to insufficient adult population coverage of the brood. This phenomenon of drastically declining bee/brood ratios is one of the defining symptoms of CCD.

Figure S1 Frames in deep box, two sides per frame showing example of capped brood.

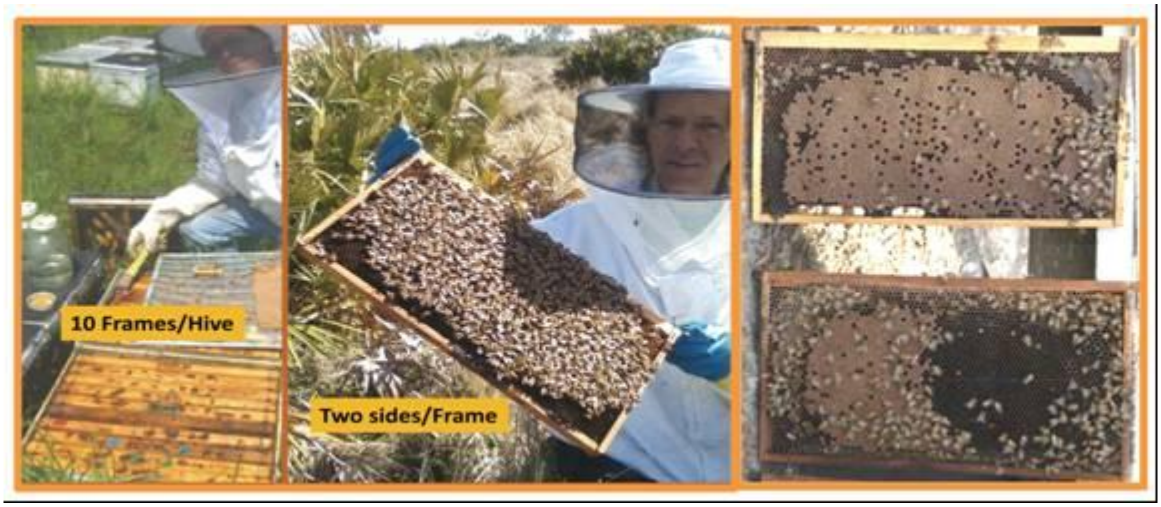

Figure S2 Adding treatment to feeding bottles.

Metal lid screws onto bottle and has three small holes from which the bees feed on the 66% sucrose solution. Hives with feeding bottles in place.

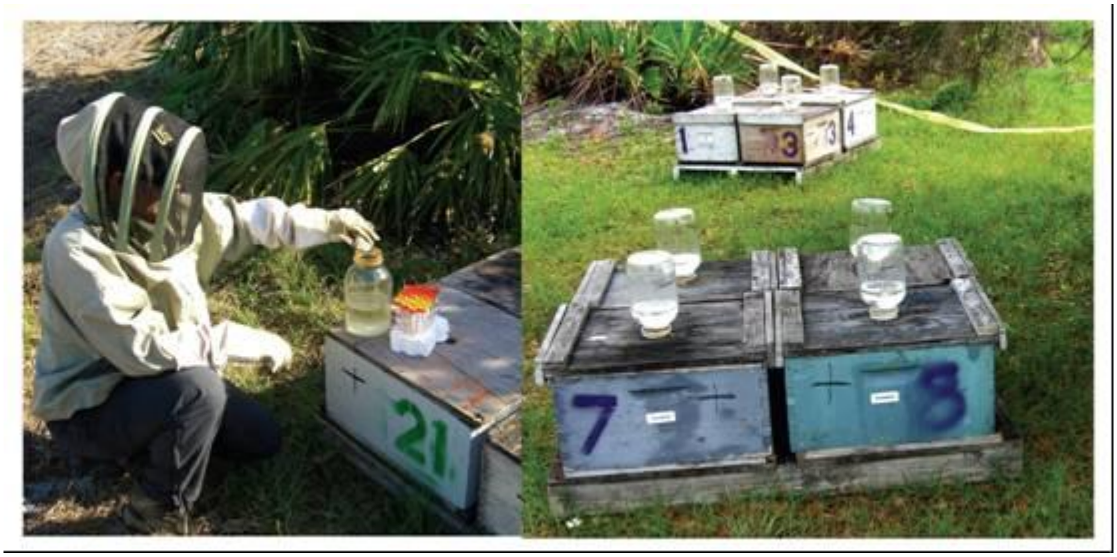

Figure S3 Counts of returning foragers on three separate dates, post treatments.

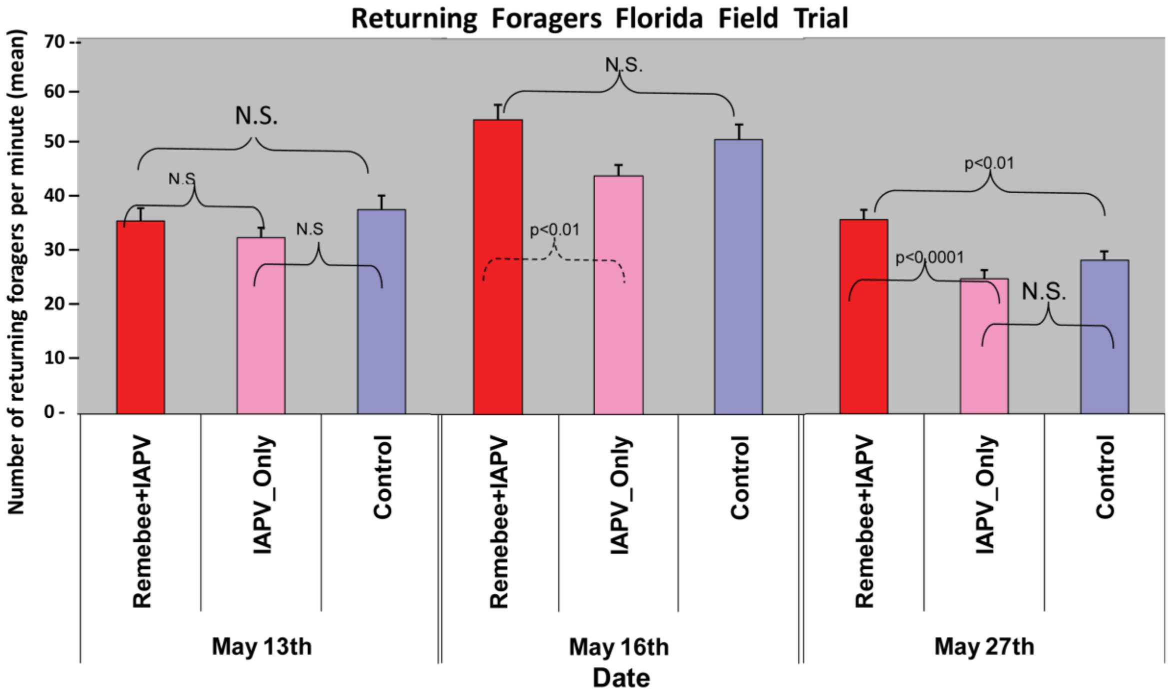

Figure S4 Bee Health. Strength of hives post treatment of Remebee+IAPV and IAPV.

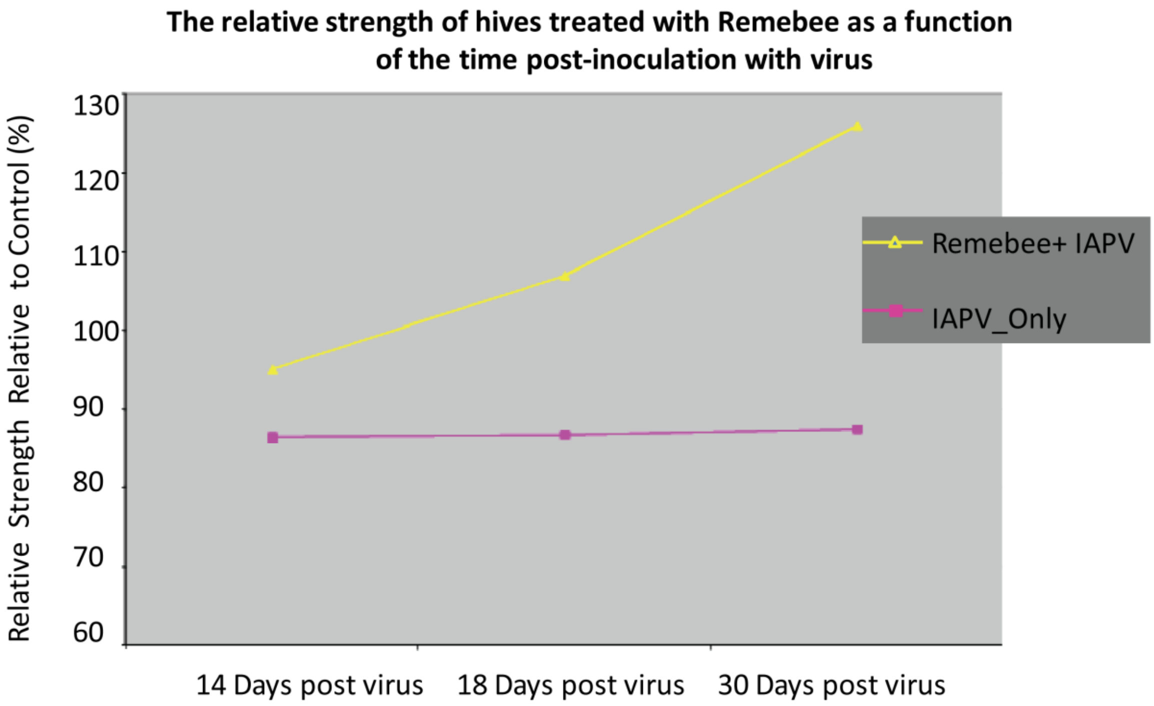

Supplement: Supporting Information S1 — Bee hives, feeding unit and field set up. (0.43 MB PDF) [file ppat.1001160.s001.pdf]
