# Supplementary material for: Large-Scale Field Application of RNAi Technology Reducing Israeli Acute Paralysis Virus Disease in Honey Bees (Apis mellifera, Hymenoptera: Apidae)
Source: PLoS Pathog. 2010 Dec 23;6(12):e1001160. doi: 10.1371/journal.ppat.1001160 (PMC3009593; doi:10.1371/journal.ppat.1001160)
Supplement: Supporting Information S2 — Detection of siRNAs in bee samples. (0.99 MB PDF) [file ppat.1001160.s002.pdf]

Large-scale field application of RNAi technology reducing Israeli Acute Paralysis Virus disease in honey bees (*Apis mellifera*, Hymenoptera: Apidae)

Wayne Hunter[1*], James Ellis[2], Dennis vanEngelsdorp[3*], Jerry Hayes[4], Dave Westervelt[4], Eitan Glick[5], Michael Williams[3], Ilan Sela[6], Eyal Maori[6], Jeff Pettis[7], Diana Cox-Foster[3], Nitzan Paldi[5*]

Supporting Information S2:- Detection of siRNAs in sampled bees in trials done in Florida and California over the winter of 2009-10

The objective of this test was to find Remebee DICER metabolites (siRNAs) in bees in the Remebee treatment groups and potentially in naturally IAPV infected bees. This corresponds to dsRNA homologous to IAPV sequences that hybridizes to the specific probe. This is a “Northern” blotting assay, which is based on the hybridization of a fluorescence probe, which hybridizes with Remebee^{TM} sequences in bees' RNA extract. This northern blotting assay **does not** distinguish between Remebee samples and IAPV sequences that are homologous to Remebee.

Samples for virus presence analysis were taken before IAPV introduction and 14 days after the IAPV introduction date. Samples were be taken from each study group, and consisted of 10 bees randomly sampled from 6 hives in each group (60 total per treatment group). The bees were either placed in RNAsafe (Ambion) to preserve the RNA from degradation, or placed on ice and immediately transferred and kept in a -80°C freezer until processed.

Procedure: Note: Detailed Materials and Methods beyond results section.

A) The **Green** group of samples represents IAPV inoculated non-Remebee-I treated bees

B) The **Red** group represents IAPV inoculated Remebee-I treated bees, once weekly.

C) The **Blue** group represents IAPV inoculated Remebee-I treated bees, once monthly.

Table S1. Tables 1-4 Key to samples (correspond to gels 1-4 below).

Florida Bees (Labels for Gels 1, 2)

	Gel #1		
Well		Label	Hive #
1	FL 12/16/2009	13	R 54A
2	FL 12/16/2009	14	R 23A
3	FL 12/16/2009	15	R 24A
4	FL 12/16/2009	16	R 53A
5	FL 12/16/2009	17	R 40A
6	90026, 90020	MR	MR 26,20
7	1192, 1194	PR	29,27
8	FL 12/16/2009	19	G 7A
9	FL 12/16/2009	20	G 19A
10	FL 12/16/2009	21	G 30A
11	FL 12/16/2009	23	G 49A
12	FL 12/16/2009	24	G 69A

	Gel#2		
Well		Hive #	Label
1	FL 01/06/2010	R 54	30
2	FL 01/06/2010	R 23	25
3	FL 01/06/2010	R 24	26
4	FL 01/06/2010	R 53	29
5	FL 01/06/2010	R 40	28
6		MR	MR 26,21
7		PR	29,27
8	FL 01/06/2010	G 7	36
9	FL 01/06/2010	G 19	31
10	FL 01/06/2010	G 30	35
11	FL 01/06/2010	G 49	34
12	FL 01/06/2010	G 69	32

California Bees (Labels for Gels 3, 4)

	Gel #3		
Well		Label	Hive #
1	CA11/25/2009	R61	
2	CA11/25/2009	R62	
3	CA11/25/2009	R63	
4	CA11/25/2009	G67	
5	CA11/25/2009	G68	
6	CA11/25/2009	G69	
7	CA11/25/2009	G70	
8	CA11/25/2009	B73	
9	CA11/25/2009	B74	
10	CA11/25/2009	B75	
11	90026, 90020	MR	MR 26,20
12	1192, 1194	PR	29,27

	Gel #4		
Well		Label	Hive #
1	CA12/09/2009	R79	
2	CA12/09/2009	R80	
3	CA12/09/2009	R81	
4	CA12/09/2009	G85	
5	CA12/09/2009	G86	
6	CA12/09/2009	G87	
7	CA12/09/2009	G88	
8	CA12/09/2009	B91	
9	CA12/09/2009	B92	
10	CA12/09/2009	B93	
11	90026, 90020	MR	MR 26,20
12	1192, 1194	PR	29,27

Results

Figure S5. Polyacrylamide Urea Gel electrophoresis

As can be seen in *Picture 1* there is a clear and even separation of bands, the marker including the primers can be clearly seen. All samples contain comparable amounts of RNA.

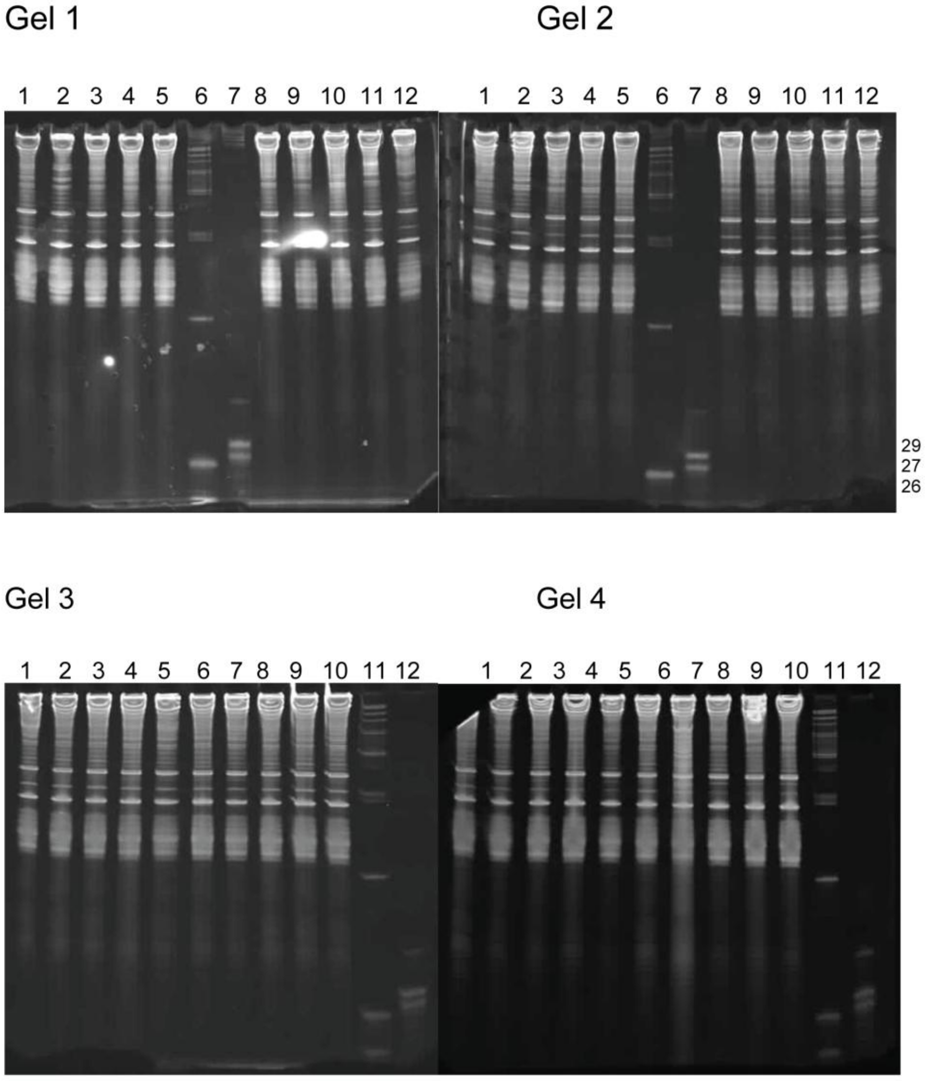

Figure **S6. Hybridization and Chemiluminescent blots of gels 1-4 as listed in Table 1-4 above.** Gels 1-2 are Florida, Lanes 1-5 (red), Lanes 8-12 (green); Gels 3-4 are California, Lanes 1-3 (red), Lanes 4-7 (green), Lanes 8-10 (blue).

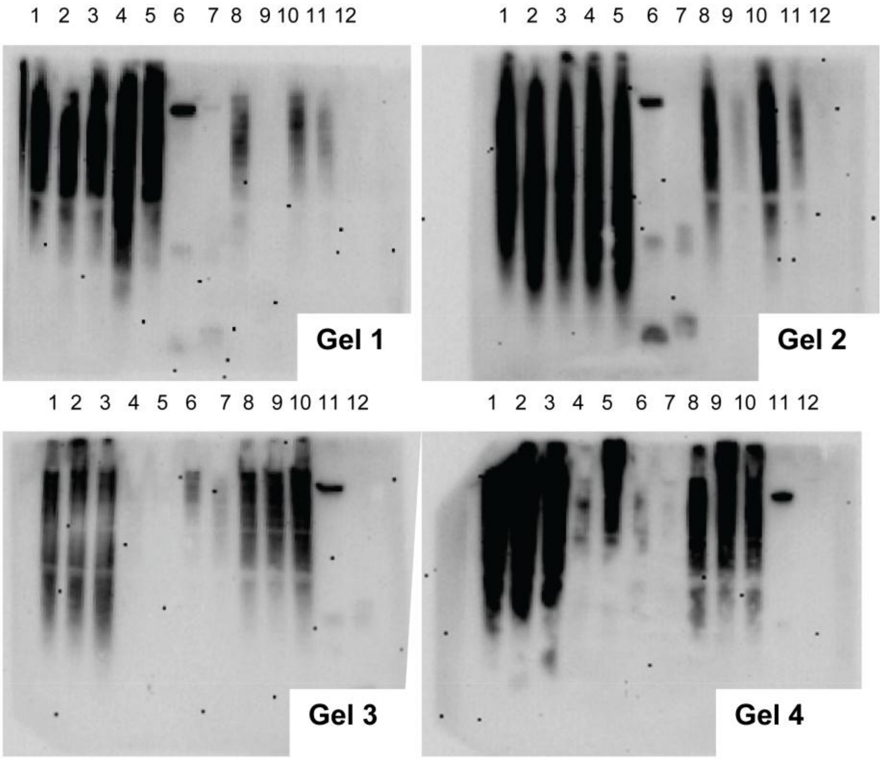

Conclusions:

1) RNA integrity and uniformity between samples was verified. Fig. S5.
2) Florida Red (Remebee weekly, Lanes 1-5) treated bees show a high level of Remebee DICER degradation products (siRNAs) Fig. S6. The sequences that hybridize with the probe are also present, albeit at lower levels, in bees from untreated control bees from Florida before and after IAPV challenge (green).
3) In California, Remebee DICER metabolites (siRNAs) are present at relatively low levels in both the weekly (red) and monthly (blue) Remebee treatments before infection. It is noteworthy that siRNAs are present in the monthly Remebee over 3 weeks after the last Remebee feeding (Gel Blots 3-4, Lanes 8-10).
4) siRNAs levels increase dramatically two weeks after challenged with IAPV in both weekly and monthly Remebee treatments but are shown to be in much lower concentrations in IAPV only challenged bees.
5) It is noteworthy that in Florida, the non-treated (green hive #'7', in lane 8, gels 1

and 2) had the highest natural siRNA levels before and after infection, and also had the highest bee population from the control group at the end-point analysis. In contrast, the two control hives that had no siRNAs either before or after infection (green hive #'19', lane 9, and green hive #69, lane12, gels 1 and 2) were either dead or were extremely weak at the end point. Comparable information from California is unfortunately not available due to the fact that bees were pooled per treatment and not per hive.

6) In samples where a relatively low level of siRNAs is present, it is easily distinguishable that the Remebee DICER metabolites are discrete bands and not a continuous smear. Fig. S6.

Additional information:

Sample preparation:

1. RNA:

3µl bee RNA sample (10µg RNA) +3ul water were mixed with 6µl 2x dye

2. Marker:

5ul marker + 1µl of 10µM each primer (26b, 20b) were mixed with 10µl 2x dye

1µl of 10µM each primer (29b, 27b) 4µl water were mixed with 10µl 2x dye

3. Samples were incubated 5min at 70°C then transferred to ice

4. 10µl (~8µg) were loaded into each well according to the loading table.

Analysis of siRNA samples by Polyacrylamide Urea Gel electrophoresis

1. Novex 15%TBE Urea Gel 12 wells were used

2. Gel run in 1xTBE

3. wells Washed carefully before 10ul samples were loaded

4. Gel Run for 90min 180V until bromophenol blue run to the bottom of the gel (xylene cyanol FF and bromophenol blue. On a 15% polyacrylamide gel, these marker dyes co-migrate with oligonucleotides with lengths of 30 and 9–10 bases respectively).

5. The Gel Soaked with Ethidium Bromide to see the RNA (picture 1-4).

Electro Blotting

1. Gel Arranged in Invitrogen and BioRad blotter with positively charge Nylon membranes

2. Run in 1XTAE buffer v for minutes with stirring.

3. Based on gel viewing after transfer; at least one of the Invitrogen blotter was very efficient (gel 3 or 4). Gel dried for 5 min

4. RNA Crosslinked in crosslinker 125mJ

Table S2. Hybridization and Chemiluminescent Detection Procedure

	Treatments	Volume	Time	Temp ºC	Lot	Performed
	**Hybridization**					
1	Incubate in **DIG Easy Hyb** buffer	10ml	30min	42		
2.	Add 100µl **preboiled probe** (10µ of CP and 10ul of IR PCR/100µl per 10 ml)	100µl/10ml		10ml per two membranes		
3	Incubate in 10 ml **DIG Easy Hyb** buffer	10ml	5-16h	42		
	**Hybridization washes**					
4	2x SSC/0.1% SDS	100ml	5	42		
5	2x SSC/0.1% SDS	100ml	5	42		
6	2x SSC/0.2% SDS	100ml	15	58		
7	2x SSC/0.2% SDS	100ml	15	50		
	**Chemiluminescent Detection**					
8	Rinse membrane briefly 1x washing buffer	100ml	5min	RT	container	
9.	Incubate in blocking solution	100ml	30min	RT	container	
10	Add 5µl Anti-Digoxigenin-AP into 50ml blocking solution = Antibody solution			50ml		
11	Incubate in Antibody solution	50ml	30min	RT	container	
12	Wash in washing buffer	100ml	15min	RT	container	
13	Wash in washing buffer	100ml	15min	RT	container	
14.	Equilibrate in detection buffer	30ml	3min	RT	container	
15	Dilute of CDP Star 1:100 indetection buffer 10µl/1ml.			1.5ml		
16	Incubate membrane in CDP 1:100 in detection buffer in a nylon bag	1.5ml	5min	RT	Nylon folder	
17.	Squeeze out excess liquid, seal the edge					
	Incubate membrane	minimum	15 min	RT		
18.	Expose 3x 20 min and 10x60 min		15- 24h	RT		

Table S3. DIG hybridization

Probe labeling: perform the following PCR reactions using PCR Dig Probe Synthesis kit cat#11_636_090:

	1	2	3	4
ddH2O	35	35	35	35
10X PCR Buffer	5	5	5	5
2mM "DIG" dNTP's	2.5		2.5	
2mM "cold" dNTP's	2.5	5	2.5	5
10µM 1191 CP PCR1 F primer	2	2		
10µM 1192 CP PCR1 R primer	2	2		
10µM 1193 IR PCR1 F primer			2	2
10µM 1194 IR PCR1 R primer			2	2
1ng\µl CP pcr0 clean template	1	1		
1ng\µl IR pcr0 clean template			1	1
enzyme mix	0.75	0.75	0.75	0.75
total	50	50	50	50

Transfer to PCR machine and run the following program: 94°C, 2 min, 94°C 30 sec, 58°C 30 sec, 72°C 30 sec, repeat 35 times; then 72°C 5 sec, hold at 4°C.

Figure S7. PCR analyses loaded at 3µl in 1% gel

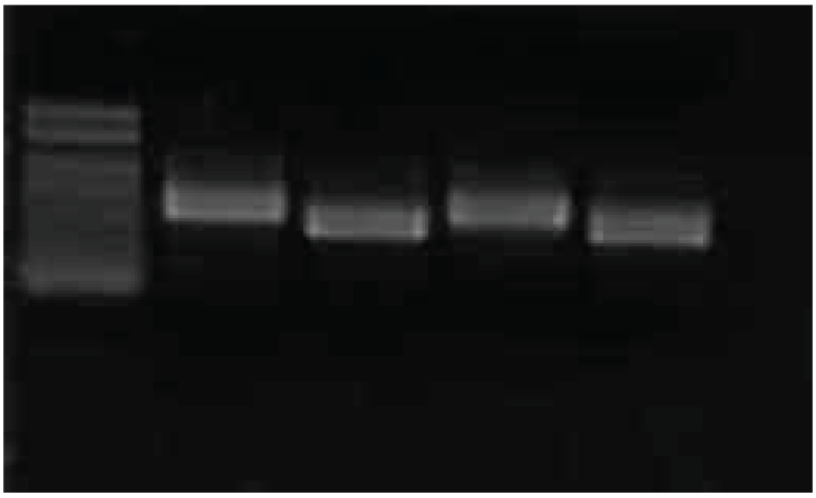

As expected slightly slower migration when using the modified UTP

Table S4. Table of primers used

Application	Primer name	Primer number	Sequence	Size	Product
CP PCR1	CPPCR1 F	V1191	TAATACGACTCACTATAGGGCGACC	25	
CP PCR1	CPPCR1 R	V1192	TAATACGACTCACTATAGGGCGATATATC	29	450
IR PCR1	IRPCR1 F	V1193	TAATACGACTCACTATAGGGCGAGAC	26	
IRPCR1	IRPCR1 R	V1194	TAATACGACTCACTATAGGGCGACATG	27	450
29b	CPPCR1 R	V1192	TAATACGACTCACTATAGGGCGATATATC	29	29b
27b	IRPCR1 R	V1194	TAATACGACTCACTATAGGGCGACATG	27	
26b	V90026	V90026	AGATTTGTCTGTCTCCCAGTGCACAT	26	26b
20b	V90020	V90020	GATTTGTCTGTCTCCCAGTG	20	21b
